# Correction: Broad dengue neutralization in mosquitoes expressing an engineered antibody

**DOI:** 10.1371/journal.ppat.1008545

**Published:** 2020-04-29

**Authors:** Anna Buchman, Stephanie Gamez, Ming Li, Igor Antoshechkin, Hsing-Han Li, Hsin-Wei Wang, Chun-Hong Chen, Melissa J. Klein, Jean-Bernard Duchemin, James E. Crowe, Prasad N. Paradkar, Omar S. Akbari

In Fig 1B, the columns corresponding to infection with DENV-3 and DENV-4 are duplicated and both denote the DENV-4 data. Please see the correct Fig 1 here.

**Fig 1:**
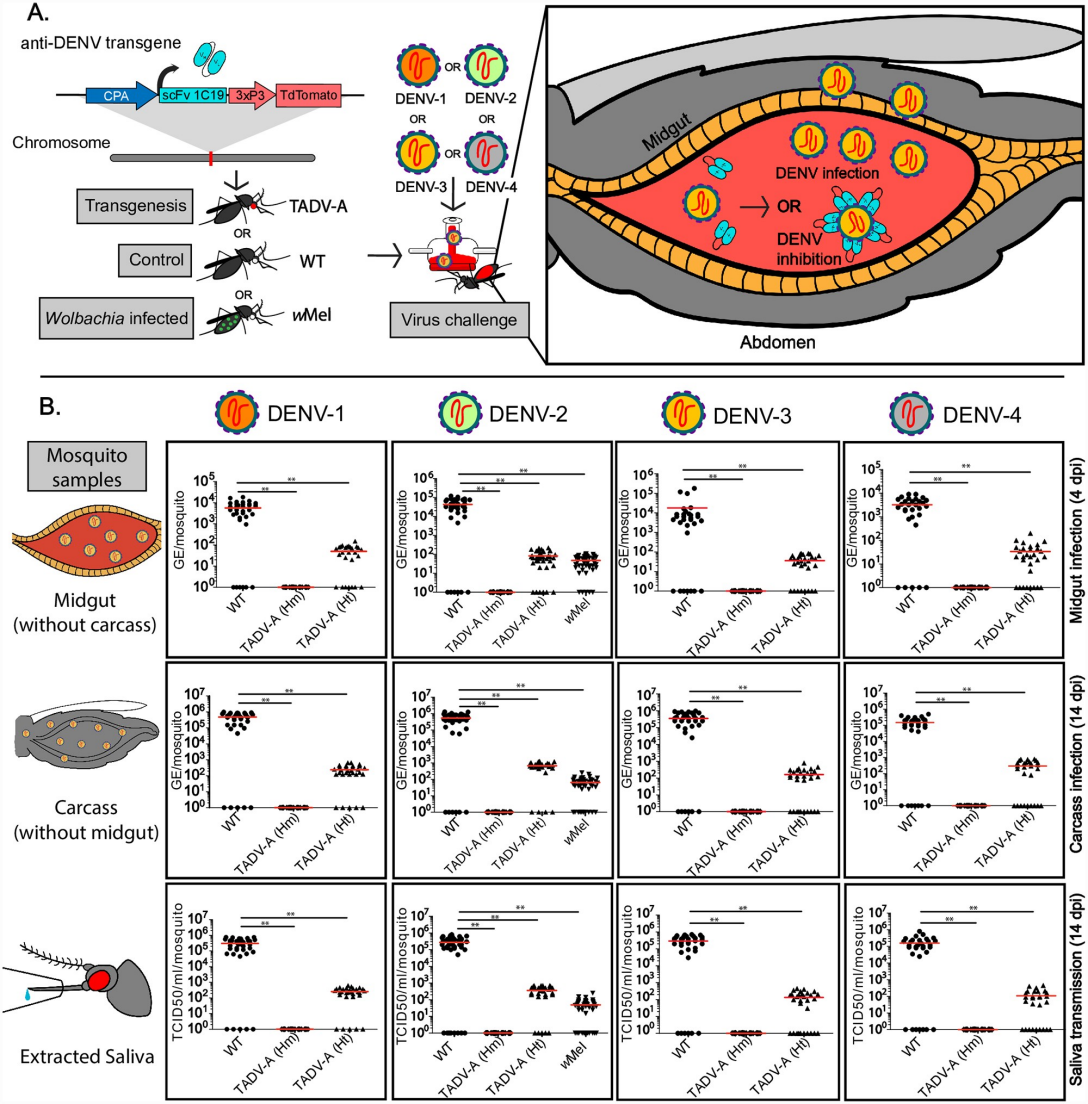
Effect of anti-dengue virus (DENV) single-chain variable fragment (scFv) on DENV titers of TADV-A, Wolbachia-infected (wMel), and wildtype (WT) mosquitoes. (A) Schematic of experiment. TADV-A mosquitoes were generated via transgenesis with the anti-DENV construct, and TADV-A, wMel, and WT mosquitoes were then challenged with a blood meal infected with one of four DENV serotypes (DENV-1, isolate ET243; DENV-2, isolate ET300; DENV-3, isolate ET209; or DENV-4, isolate ET288). After the infected blood meal enters the mosquito midgut, there are two potential outcomes: in the first (applies for all tested strains), the virus replicates and disseminates past the midgut to become transmissible; in the second (applies to TADV-A mosquitoes), the anti-DENV transgene expresses scFv antibodies in the midgut that bind to the virus and neutralize it. (B) Plots depicting viral titers. To determine if the anti-DENV transgene confers resistance to all four DENV serotypes, we determined viral titers in extracted midguts, carcasses, and saliva from WT, TADV-A (homozygous [Hm] and heterozygous [Ht]), and wMel infected mosquitoes. Viral genome equivalents (GE) from mosquito midguts (at 4 days post infection [dpi]) and carcass (at 14 dpi) were determined using RT-qPCR and calculated using previously published methods. Viral titers in the saliva were determined using the median tissue culture infective dose (TCID50) on Vero cells. For each experiment, data from three replicates is pooled. Red horizontal bars represent the mean GE/viral titer. **p < 0.001.
