# Supplementary figures and images for: Broad dengue neutralization in mosquitoes expressing an engineered antibody
Source: PLoS Pathog. 2020 Jan 16;16(1):e1008103. doi: 10.1371/journal.ppat.1008103 (PMC6964813; doi:10.1371/journal.ppat.1008103)

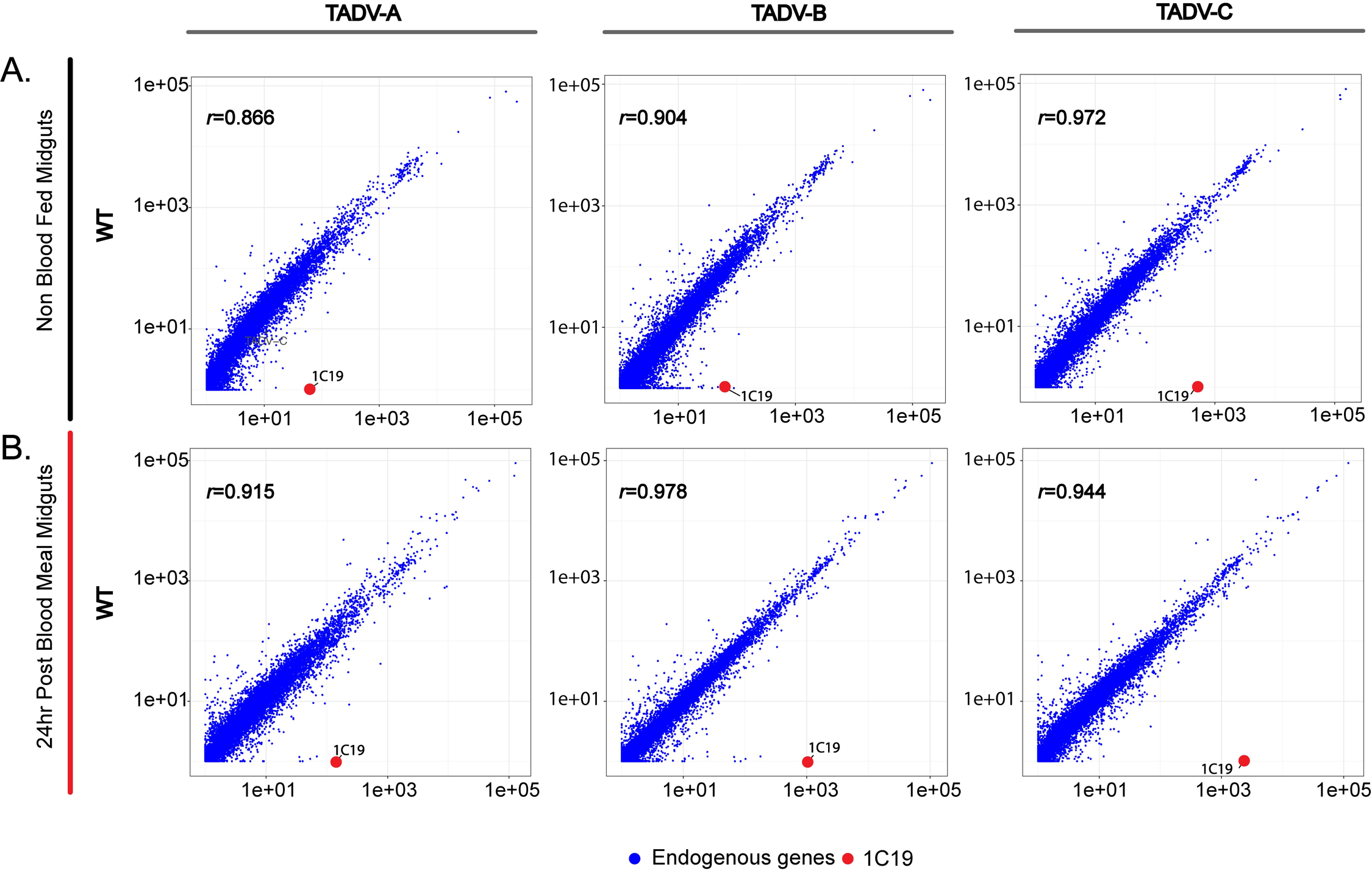

Supplement: S1 Fig — Expression correlation analyses of gene expression levels (indicated by TPM [transcripts per million] values) in dissected midgut tissues from WT or transgenic (TADV-A, -B, or -C) mosquitoes without a blood meal (A) and 24 hours after a blood meal (B). The y-axis corresponds to TPM values in WT samples, and the x-axis corresponds to TPM values in respective transgenic samples. Blue-colored points represent endogenous genes, and red-colored points represent the 1C19 scFv. Comparisons between WT and TADV samples suggest that 1C19 scFv expression is transgene-dependent and does not appear to significantly affect global expression levels of endogenous RNAs. Pearson correlation coefficients (r) between gene expression levels in WT versus transgenic samples are reported in bold in their respective graphs. (TIF) [file ppat.1008103.s001.tif]

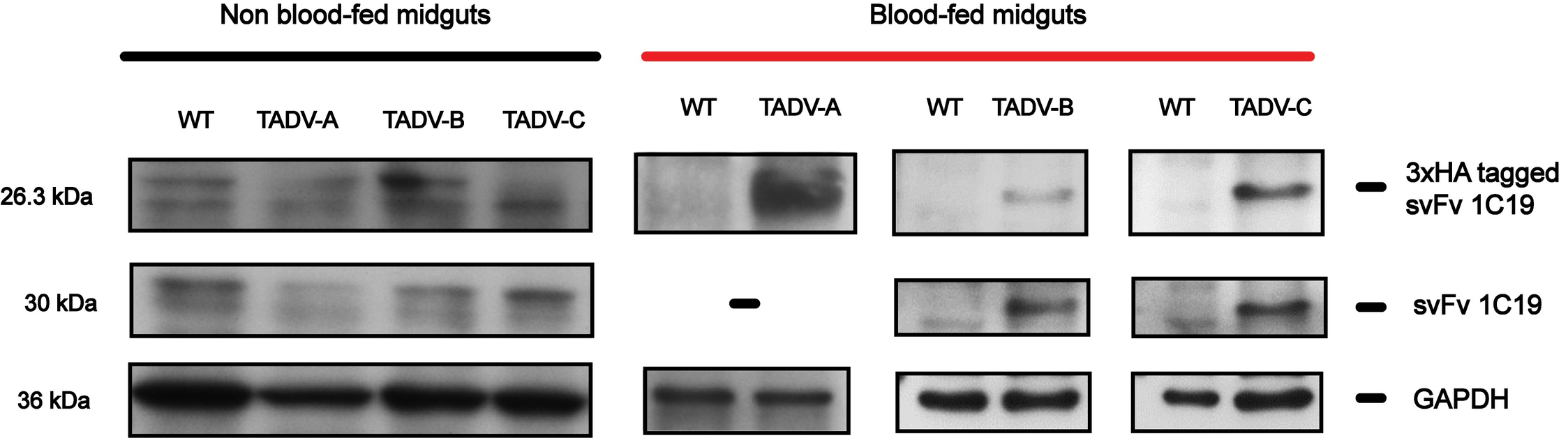

Supplement: S2 Fig — Western blots were carried out utilizing a custom antibody specific for the 1C19 scFv protein, as well as an antibody to recognize the 3xHA tag, on dissected midgut tissues from non-blood-fed or 16-hour post-blood-meal WT or TADV-A, TADV-B, or TADV-C mosquitoes. An anti-GAPDH antibody was used as a control. The presence of a 26.3-kDa band confirms the expression of the 1C19 scFv protein in transgenic, but not in WT, mosquito midgut samples. The presence of a 30 kDa band indicates the presence of the 3xHA tag in TADV-B and TADV-C but not in WT or TADV-A mosquitoes. (TIF) [file ppat.1008103.s002.tif]

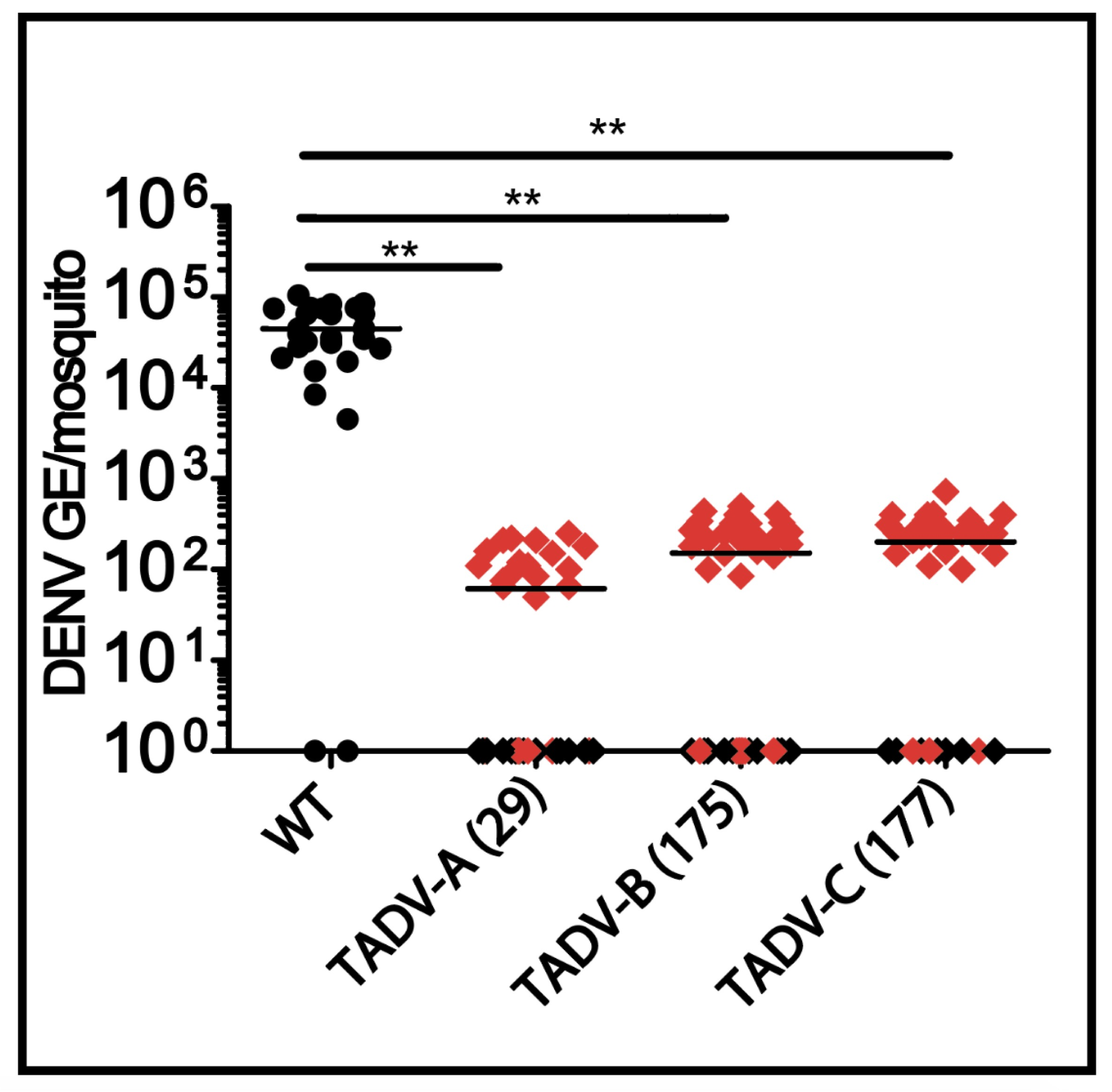

Supplement: S3 Fig — DENV GE in WT and transgenic mosquito lines (TADV-A, TADV-B, or TADV-C) following a blood meal infected with DENV-2 (ET300 strain) are shown. DENV GE from mosquito midguts (at 4 dpi) of WT or transgenic mosquitoes were determined using real-time qPCR and calculated using previously published methods. Circles represent WT mosquitoes; black diamonds represent anti-DENV homozygous transgenic mosquitoes; red colored diamonds represent anti-DENV heterozygous transgenic mosquitoes. Horizontal bars represent the mean viral titer. The Mantel-Cox test was used for statistical analysis. **p < 0.001. (TIF) [file ppat.1008103.s003.tif]

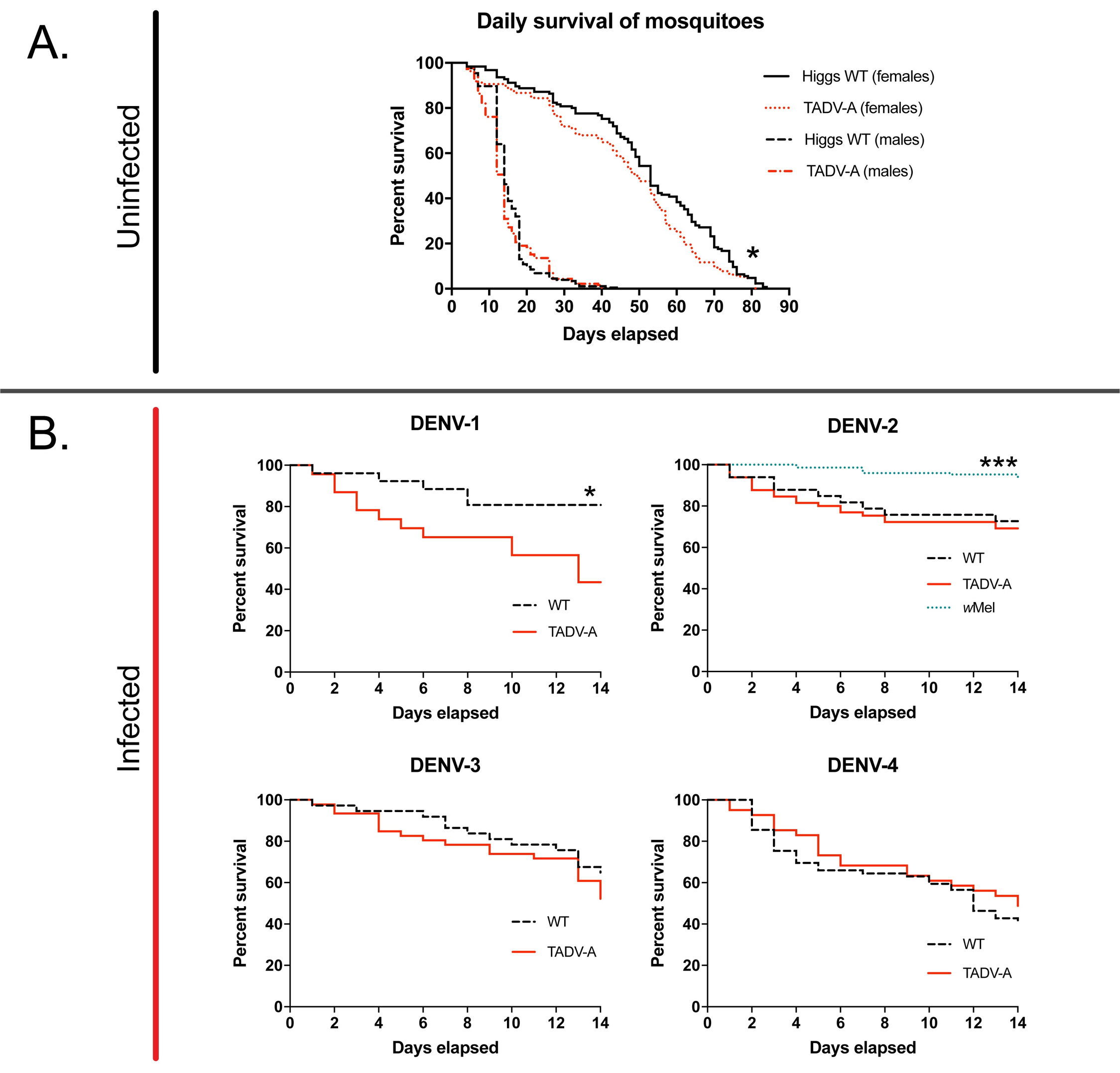

Supplement: S4 Fig — The x-axis indicates the number of days elapsed after the start of the experiment, and the y-axis indicates the percent of mosquitoes surviving on each elapsed day. (A) For the uninfected panel, where the survivorship curves for WT and TADV-A male and female mosquitoes are shown separately, each line represents the accumulated results of 120–180 adult mosquitoes combined from 3 biological replicates. Significant differences in survivorship were observed between WT and TADV-A females, with WT females surviving, on average, 4.5 days longer (p ≤ 0.01). (B) For the infected panels, WT, wMel, and TADV-A females were given a blood meal infected with DENV-1, DENV-2, DENV-3, or DENV-4 (as indicated on respective plot titles). The survivorship of infected mosquitoes was determined over the course of 14 days (the time it takes for the virus to disseminate past the midgut and eventually become transmissible). No significant differences in survivorship were found between WT and TADV-A mosquitoes when infected with DENV-3 and DENV-4, but significant differences were observed upon infection with DENV-1, with more WT mosquitoes (80%) surviving at 14 dpi than TADV-A mosquitoes (~40%; p ≤ 0.01). When infected with DENV-2, more wMel mosquitoes (>90%) survived at 14 dpi compared to WT and TADV-A mosquitoes (both ~70%; p < 0.0001). The Mantel-Cox test was used to determine statistical significance. *p ≤ 0.01, ***p < 0.0001. (TIF) [file ppat.1008103.s004.tif]
