# Supplementary material for: Broad dengue neutralization in mosquitoes expressing an engineered antibody
Source: PLoS Pathog. 2020 Jan 16;16(1):e1008103. doi: 10.1371/journal.ppat.1008103 (PMC6964813; doi:10.1371/journal.ppat.1008103)
Supplement: S1 Table — DENV-2 GE are shown below for WT, TADV-A, TADV-B, and TADV-C mosquito lines following a blood meal infected with the DENV-2 ET300 strain. Midgut samples were collected 4 dpi, and GE were determined using real-time RT-qPCR and calculated using previously published methods. (DOCX) [file ppat.1008103.s005.docx]

**S1 Table.** Effect of the anti-DENV scFv on DENV-2 GE in three independent mosquito lines. DENV-2 GE are shown below for WT, TADV-A, TADV-B, and TADV-C mosquito lines following a blood meal infected with the DENV-2 ET300 strain. Midgut samples were collected 4 dpi, and GE were determined using real-time RT-qPCR and calculated using previously published methods.

| **Mosquito strain** | **GE of virus RNA/mosquito midgut at 4 dpi** | **Virus-positive mosquitoes/ Total mosquitoes (% infection rate)** |
| --- | --- | --- |
| WT | 4.5 x 10^4^ | 24/26 (92.3%) |
| TADV-A | 6.1 x 10^1^ | 17/37 (45.9%) |
| TADV-B | 1.5 x 10^2^ | 25/40 (62.5%) |
| TADV-C | 2.0 x 10^2^ | 25/35 (71.4%) |
