# Supplementary material for: Broad dengue neutralization in mosquitoes expressing an engineered antibody
Source: PLoS Pathog. 2020 Jan 16;16(1):e1008103. doi: 10.1371/journal.ppat.1008103 (PMC6964813; doi:10.1371/journal.ppat.1008103)
Supplement: S2 Table — (DOCX) [file ppat.1008103.s006.docx]

**S2 Table**. Primers utilized to generate anti-DENV scFv used in this study.

| **Primer** | **Primer Sequence, 5’ to 3’** | **Source** |
| --- | --- | --- |
| tdTomato marker  984.1A  984.1B | GACGGTACGATCCACCGGTCGCCACCATGGTGAGCAAGGGCGAGGAGGTCATCAAAGAGT  TGGTATGGCTGATTATGATCTAGAGTCGCGGCCGCCTACTTGTACAGCTCGTCCATGCCG | Gene-  synthesized vector |
| Carboxypeptidase promoter  984.2A      984.2B | ATGGTTAATTCGAGCTCGCCCGGGGTCCTAGGGAATTCGTCAATAAAAAAATACGTTCAA  CTCCTCGCCCTTGCTCACCATGTTTAAACTTTCCCAACTAACCGATACACACTAACCTG | Genomic *Ae. aegypti* DNA |
| GFP marker  984.3A  984.3B | AGTGTGTATCGGTTAGTTGGGAAAGTTTAAACATGGTGAGCAAGGGCGAGGAGCTGTTCAC  TGATTTGTTATTTTAAAAACGATTCATTCTAGTTAATTAATTACTTGTACAGCTCGTCCATGCC | pMos[3xP3-eGFP] |
| p10 3’ UTR  984.4A  984.4B | GGACGAGCTGTACAAGTAATTAATTAACTAGAATGAATCGTTTTTAAAATAACAAAT  TCCCCGGGCGAGCTCGAATTGGCGCGCCCGGCCGTTAACTCGAATCGCTATCCAAGC | pJFRC81-10XUAS-IVS-Syn21-GFP-p10 |
| G(4)-S linker  984B.C1  984B.C2 | TTCGGTCAGGGAACCAAAGTGGATATTAAGGGCGGTTCCGGTGGCAGCGGCGGCTCCGGTGGCAGCTACCCCTACGACGTGCCCG  TGTTATTTTAAAAACGATTCATTCTAGTTAATTAATCAGGCGTAATCTGGCACGTCGTAC | *ninaE*[SBP-His] vector |
