# Supplementary material for: Broad dengue neutralization in mosquitoes expressing an engineered antibody
Source: PLoS Pathog. 2020 Jan 16;16(1):e1008103. doi: 10.1371/journal.ppat.1008103 (PMC6964813; doi:10.1371/journal.ppat.1008103)
Supplement: S3 Table — (DOCX) [file ppat.1008103.s007.docx]

**S3 Table.** Diagnostic primers used for inverse PCR (iPCR) assays, zygosity confirmation, pan-DENV serotype detection, and *w*Mel infection confirmation.

| ***piggyBac* Primers** | | | |
| --- | --- | --- | --- |
| **Reaction** | **Primer Name** | | **Primer Sequence, 5’ to 3’** |
| 5’ (1st Round PCR) | 991.5F1 | | GACGCATGATTATCTTTTACGTGAC |
|  | 991.5R1 | | TGACACTTACCGCATTGACA |
| 5’(2nd Round PCR) | 991.5F2 | | GCGATGACGAGCTTGTTGGTG |
|  | 991.5R2 | | TCCAAGCGGCGACTGAGATG |
| 3’ (1st Round PCR) | 991.3F1 | | CAACATGACTGTTTTTAAAGTACAAA |
|  | 991.3R1 | | GTCAGAAACAACTTTGGCACATATC |
| 3’ (2nd Round PCR) | 991.3F2 | | CCTCGATATACAGACCGATAAAAC |
|  | 991.3R2 | | TGCATTTGCCTTTCGCCTTAT |
| **Zygosity Primers** | | | |
| **Reaction** | **Line** | **Primer Name** | **Primer Sequence, 5’ to 3’** |
| Forward primer | TADV-A | 1018.S19 | CCAAGTCGTCAACTTGTGACTG |
|  | TADV-B | 1018.S73 | TAAAAACATAAACAACGACC |
|  | TADV-C | 1018.S80 | GCTCTCACCGTTGAAAATTTAT |
| Reverse primer | TADV-A | 1018.S21 | GGACCCAATTGTTACTACACCTATTTC |
|  | TADV-B | 1018.S74 | AAAATAAATCATTCTGAGTTTGT |
|  | TADV-C | 1018.S82 | CAGGGTCCTAAAATGTTCAATG |
| **DENV RT-qPCR Primers** | | | |
| **Primer name** | | **Primer Sequence, 5’ to 3’** | |
| DENV-F | | ACGTGCACACATGGACAGA | |
| DENV-R | | ACTGAGCGGATTCCACAAA | |
| ***w*Mel Primers** | | | |
| **Primer name** | | **Primer Sequence, 5’ to 3’** | |
| *w*Mel-F | | CAAATTGCTCTTGTCCTGTGG | |
| *w*Mel-R | | GGGTGTTAAGCAGAGTTACGG | |
