# Supplementary material for: Broad dengue neutralization in mosquitoes expressing an engineered antibody
Source: PLoS Pathog. 2020 Jan 16;16(1):e1008103. doi: 10.1371/journal.ppat.1008103 (PMC6964813; doi:10.1371/journal.ppat.1008103)
Supplement: Attachment — Submitted filename: PLOS Pathogens Rebuttal.docx [file ppat.1008103.s011.docx]

September 6th, 2019

Dear PLOS Pathogens Editors,

Please find enclosed our revised manuscript entitled, “Broad Dengue Neutralization in Mosquitoes Expressing an Engineered Antibody” for publication as an article in PLOS Pathogens. We greatly appreciate the reviewers' comments and thank them for their insightful suggestions, as we believe they have significantly improved our manuscript. We have constructively addressed each of their points below (reviewer comments in black, our response in blue text, and any significant textual changes pasted below) and we are looking forward to seeing this important work in press at PLOS Pathogens.

Thanks in advance for your reconsideration.

Sincerely,

Omar S. Akbari, Ph.D., Associate Professor, Section of Cell and Developmental Biology, University of California, San Diego, La Jolla, California 4430 Bonner Hall, La Jolla CA,  92093, (858) 246-0640, [oakbari@ucsd.edu](mailto:oakbari@ucsd.edu)

~~~~~~~~~~~~~~~~~~~~~~~~~~~~~~~~~~~~~~~~~~~~~~~~~~~~~~~~~~~~~~~~~~~~~~~~

Reviewer's Comments

**Part I - Summary**

Reviewer #1: The authors present new genetically engineered Ae. aegypti strain expressing a highly and broadly cross-neutralizing single chain antibody against DENV. They demonstrate through a series of experiments that these engineered mosquitoes can effectively (100%) inhibit the infection, dissemination and transmission of all four serotypes of DENV. Further, they demonstrate that their strain is even more effective than Wolbachia, which is currently the gold standard for DENV inhibition. Overall this is a very exciting and impactful manuscript. Below are my critiques.

Reviewer #2: The study by Buchman et al generated Aedes aegypti mosquitoes that produce a single chain antibody that is capable of neutralizing all four strains of Dengue virus. The paper is well written and contains necessary controls and experiments for the conclusions they are drawing. They engineered the antibody expression to coincide with blood meal digestion to limit expression and find it had little impact on mosquito fitness. Mosquitoes homozygous for the antibody appear completely refractory for virus whereas heterozygotes tested positive for virus, but contained significantly lower quantities of virus. They include Wolbachia infected mosquitoes to compare their results with other well-established strategies to decrease mosquito competence for Dengue transmission. They find that the heterozygotes carrying the antibody decrease viral quantities similar to Wolbachia infected mosquitoes and homozygotes are more effective. While outside of this particular study, I cannot help but wonder how quickly Dengue will be able to escape neutralization from a single antibody. This is a very provocative study demonstrating a new method to decrease dengue spread. Future work would require incorporating antibody expression with a potent gene drive.

Reviewer #3: This is a very elegant study where transgenic Aedes aegypti expressing an engineered single-chain variable fragment derived from a broadly neutralizing dengue virus human monoclonal antibody are refractory to all four dengue virus serotypes. As such the study can be considered a significant advance that paves the way to develop transgenic mosquito-based dengue control. One weakness is the lack of basic biological insight.

Reviewer #4: Strengths: This paper by Buchman et al builds on previous work by the group where they engineered miRNAs in Aedes aegypti for resistance to DENV1-4. In the paper under review, they have engineered Mab 1C19 as a single-chain variable fragment (scFv) to build an effector transgene for resistance to multiple DENV serotypes. The key innovative aspect of this work is the development of the scFv from IC19 since the monoclone has broad neutralzing activity against the four serotypes. This type of approach (targeting all four serotypes) has been a 'holy grail' of geneticists/arbovirologists interested in designing Aedes aegypti for resistance to DENVs and furthers population replacement strategies/approaches. This research group also has a continued interest in developing antiviral transgenes in the context of gene drive so this might all come together. An important feature of this work is the IC19 scFv expression levels are increased with homozygosity generating apparent complete resistance to DENV1-4. It was a nice touch to compare DENV resistance of TADV-A vs wMel Wolbachia infected mosquitoes. The paper is well written and follows a logical experimental approach for developing transgenesis and characterizing transgene efficiency and a DENV 1-4 resistance phenotype.

Weaknesses: The weaknesses are minor. However, the approach brings up two points. First, in theory minor changes in the viral genome at the target site could reduce or ablate resistance by altering the ability of the scFv to neutralize. The authors discussed this in their paper and thought they could manage this outcome through selection of other scFVs transgenes, but these may run into the same problem. Nevertheless this approach may drive evolution/selection of viruses that fail to interact with these transgene effectors. Perhaps building transgenics with multiple transgenes may mitigate the ability of DENVs to adapt to any one transgene and designing a mosquito with a combination of effector gene approaches may be a future goal.

We agree with the reviewer that the possibility of resistance arising is an important consideration that could possibly addressed by combining different types of DENV refractory genes, and have further modified the text to reflect this:

“Indeed, several studies have shown that, in some contexts, DENV can rapidly evolve resistance in response to neutralizing antibodies (Zou et al. 2012; Lai et al. 2007), and this may be especially likely in the TADV-A heterozygotes described in this study. However, this potentially can be managed through the selection of antibodies with mechanisms/epitopes that minimize the chance of evolved resistance and the use of a combination of distinct anti-DENV antibodies (many of which have been described, e.g.,(Zou et al. 2012; Yamanaka, Kotaki, and Konishi 2013; Budigi et al. 2018; Lai et al. 2007; Smith et al. 2013)), as well as a combination of antibody and non-antibody based DENV refractoriness transgenes (e.g., (Franz et al. 2006; Mathur et al. 2010; Yen et al. 2018; Jupatanakul et al. 2017)).”

Second, the fitness of the TADV-A may be further improved by introgressing the scFv transgene into a field-collected (true wild-type) strain of Aedes aegypti since the progenitor HWE strain contains recessive lethals that compromise fitness. However, I view these as minor issues for this paper. Please address.

We thank the reviewer for this observation, and have modified the text to reflect this point:

“Additionally, while we observed no significant reduction in multiple fitness parameters in transgenic mosquitoes when compared to WT, some differences in fitness were observed, and more extensive analyses on fitness of both infected and uninfected transgenic heterozygotes and homozygotes (possibly after introgression with a field-collected mosquito strain) would have to be performed before use of such mosquitoes in the field.”

**Part II – Major Issues: Key Experiments Required for Acceptance**

Reviewer #3: The authors could have elaborated more on biological aspects, such as the RNAseq data (unlikely that no endogenous mosquito gene were not regulated).

We thank the reviewer for this comment, and agree that this is an important point to include. Therefore, we have performed additional analyses on the RNAseq data (as detailed in the Methods section and newly added Supplementary Tables S5 and S6), and have modified the text to reflect this additional analysis:

“Importantly, while there were some changes in expression of some genes in transgenic mosquitoes when compared to WT, these represented a small fraction of the genome and, with a few exceptions, did not appear to be consistent between the three transgenic lines (Supplementary Table 5 and S6). This suggests that the 1C19 scFv expression does not affect gene-expression patterns in a major, global way (Figure S1, Supplementary Tables S4-S6).”

The authors should also elaborate on the fitness data that did show some interesting patterns especially for dengue virus 1.The comparison between the transgenic mosquitoes and Wolbachia only involved one virus strain (a lab strain) so one should be cautious with the interpretation of that data.

We thank the reviewer for these comments, and have modified the text to reflect these points:

“Additionally, while we observed no significant reduction in multiple fitness parameters in transgenic mosquitoes when compared to WT, some differences in fitness were observed, and more extensive analyses on fitness of both infected and uninfected transgenic heterozygotes and homozygotes (possibly after introgression with a field-collected mosquito strain) would have to be performed before use of such mosquitoes in the field.”

**Part III – Minor Issues: Editorial and Data Presentation Modifications**

Please use this section for editorial suggestions as well as relatively minor modifications of existing data that would enhance clarity.

Reviewer #1: 1. The following excerpt is a run-on sentence and should be changed. Line numbering would have been helpful.

“Moreover, releases of mosquitoes artificially infected with the intracellular endosymbiont Wolbachia, which can make infected males incapable of successfully mating with uninfected females in an SIT-like manner and can inhibit mosquito infection with pathogens such as DENV and ZIKV(Aliota et al., 2016;Walker et al., 2011), have also been carried out to either suppress mosquito populations or make them less likely to transmit pathogens, and may hold promise for reducing incidence of disease(Moreira et al., 2009; Schmidt et al., 2017).”

We appreciate the reviewer pointing out this grammatical error, and have corrected the text to remove it.

2. A description of the carboxypeptidase promoter should be presented. Presumably, it is constitutively expressed and only translated following a bloodmeal? This should be included somewhere in the manuscript.

We thank the reviewer for pointing out this oversight, and have included a brief description of the carboxypeptidase promoter in the text:

“Previous findings determined that the CP promoter induces enhanced transcription of *Aedes aegypti* CPA mRNA after a blood meal and a somewhat moderate expression in sugar-fed mosquitoes [(Edwards et al. 2000)](https://paperpile.com/c/jgjUht/X9US).”

3. It is unclear where this scFv is expressed. Is it secreted, membrane bound or cytosolic? There was no mention of its localization. If it is secreted how does the antibody bind the virus prior to infection? Presumably, transcripts will be translated upon bloodfeeding (see above comment), but temporally how does this align with the rate of midgut infection? Can the antibodies still block infection after internalization into early endosomes? I am not suggesting this paper needs to be converted into a mechanistic paper, but it should discuss these questions.

We thank the reviewer for this comment, and have provided more information about the expression and localization of the antibody and a potential mechanism of how the scFv is able to bind to the virus to prevent replication.

In particular, the text now reads:

“The scFv is secreted into the epithelium of the posterior midgut in mosquitoes [(Edwards et al. 2000)](https://paperpile.com/c/jgjUht/X9US). “

“Given previous characterizations of the 1C19 scFv antibody, we presume that it achieves this refractoriness because, when it is secreted into the epithelium of the posterior midgut in mosquitoes (Edwards et al. 2000), it binds to the exposed fusion loop of DENV and inhibits the virus particle from releasing its genome into the cytoplasm for replication.”

4. The first paragraph of the discussion discusses how heterozygous mosquitoes could be sufficient to halt transmission due to their reduction in transmission. This may or may not be true which is acknowledged; however, if this argument that heterozygous mosquitoes could be effectively used in nature if needed than it would have been nice to see the fitness studies expanded to include the TADV-A heterozygotes.

We thank the reviewer for this comment, and agree that, given more time and resources, a fitness characterization of the TADV-A heterozygotes would be interesting. However, given that we logically expected homozygous TADV-A-bearing mosquitoes to be less fit than their heterozygous counterparts since they expressed more transgenes (double), we reasoned that the fitness data on the homozygotes could be extrapolated to the heterozygotes, given that the fitness effects anticipated in the latter should be no greater than those seen in the former.

5. The authors discuss the possibility of escape mutants in the discussion. I don’t think this is a major concern in the homozygotes because they demonstrated sterilizing immunity; however, this could be important in heterozygotes.

We agree with the reviewer’s observation on this point, and have modified the text to reflect this:

“Indeed, several studies have shown that, in some contexts, DENV can rapidly evolve resistance in response to neutralizing antibodies (Zou et al. 2012; Lai et al. 2007), and this may be especially likely in the TADV-A heterozygotes described in this study. However, although this can be managed through the selection of antibodies with mechanisms/epitopes that minimize the chance of evolved resistance and the use of a combination of distinct anti-DENV antibodies, many of which have been described (e.g.,(Zou et al. 2012; Yamanaka, Kotaki, and Konishi 2013; Budigi et al. 2018; Lai et al. 2007; Smith et al. 2013)).”

Reviewer #2: Minor:

What was the concentration of virus in the blood-meal? I was unable to find this information in the manuscript.

We thank the reviewer for pointing out this oversight. The final concentration of virus in the blood-meal was 1x10^5 TCID50/ml for all 4 serotypes. This has been added to the Methods section, which now reads:

“Briefly, female mosquitoes were challenged with an infected blood meal (1x10^5 TCID50 /mL) through membrane feeding using chicken blood and skin.”

Sup Fig 1: Western blots for the Non blood-fed midguts should be run on the same gel as the positives to demonstrate relative expression levels. Running the samples on multiple gels makes things harder to evaluate and potentially makes the background bands more apparent.

While we agree with the reviewer that running all samples on the same gel would have been ideal, we feel that the presented blot image is sufficient to qualitatively depict whether or not protein was present. With respect to quantitative analyses, we posit that the RNA-seq data in Supplementary Table 4 give a more accurate quantification (in terms of TPM) of the expression levels among samples than a blot would be able to provide.

References

[Edwards, M. J., L. A. Moskalyk, M. Donelly-Doman, M. Vlaskova, F. G. Noriega, V. K. Walker, and M. Jacobs-Lorena. 2000. “Characterization of a Carboxypeptidase A Gene from the Mosquito, Aedes Aegypti.” *Insect Molecular Biology* 9 (1): 33–38.](http://paperpile.com/b/jgjUht/X9US)

[Rey, Félix A., Karin Stiasny, Marie‐christine Vaney, Mariano Dellarole, and Franz X. Heinz. 2018. “The Bright and the Dark Side of Human Antibody Responses to Flaviviruses: Lessons for Vaccine Design.” *EMBO Reports*. https://doi.org/](http://paperpile.com/b/jgjUht/nnz2)[10.15252/embr.201745302](http://dx.doi.org/10.15252/embr.201745302)[.](http://paperpile.com/b/jgjUht/nnz2)

[Smith, Scott A., A. Ruklanthi de Alwis, Nurgun Kose, Eva Harris, Kristie D. Ibarra, Kristen M. Kahle, Jennifer M. Pfaff, et al. 2013. “The Potent and Broadly Neutralizing Human Dengue Virus-Specific Monoclonal Antibody 1C19 Reveals a Unique Cross-Reactive Epitope on the Bc Loop of Domain II of the Envelope Protein.” *mBio* 4 (6): e00873–13.](http://paperpile.com/b/jgjUht/h3Wy)
